# Two *Plasmodium vivax* hypnozoite-expressed RNA-binding proteins inhibit liver stage replication

**DOI:** 10.21203/rs.3.rs-8080228/v1

**Published:** 2025-11-28

**Authors:** Kim Chi Vo, Riëtte van Biljon, Gigliola Zanghi, Bryan Zavala-Martinez, William Betz, Ashley M. Vaughan, Heather J Painter, Stefan H I Kappe

**Affiliations:** 1 Center for Global Infectious Disease Research, Seattle Children’s Research Institute, Seattle, WA, USA.; 2 Department of Pediatrics, University of Washington, Seattle, WA, USA.; 3 Department of Global Health, University of Washington, Seattle, WA, USA.; 4 Division of Bacterial, Parasitic, and Allergenic Products, Office of Vaccines Research and Review, Center for Biologics Evaluations and Research, Food and Drug Administration, Silver Spring, MD, USA.

## Abstract

*Plasmodium vivax (Pv)* forms non-replicating liver stages called hypnozoites, which activate after primary infection, and cause relapses of symptomatic blood-stage malaria. We hypothesized that hypnozoites must actively suppress schizogony to maintain a quiescent state. Differential transcriptome prospecting idenfied two hypnozoite-expressed transcripts encoding putative RNA-binding proteins. We assessed the functional role of the two encoded proteins in *Plasmodium yoelii (Py)*, a rodent malaria parasite that naturally does not form hypnozoites. Strikingly, individual expression of each protein in *Py* liver stages blocked liver stage schizogony, with parasites remaining small and uninucleate. A screen of RNA sequences that interact with the putative RNA-binding domains of these proteins showed enrichment of distinct, highly specific motifs, indicating that these protein might block schizogony by binding RNAs containing these motifs. Our findings provide the unprecedented functional evidence for one potential molecular mechanism of hypnozoite formation. Based on their function we named these proteins IESI-1 and IESI-2 (Initiation of Exo-erythrocytic Schizogony inhibited).

## Introduction

Malaria remains a globally prevalent infectious disease caused by *Plasmodium* parasites, resulting in approximately 263 million clinical cases and 597,000 deaths across 83 endemic countries in 2023, an increase of 11 million cases compared with 2022 (World Malaria Report 2024)^1^. Among human malaria parasites, *P. falciparum* causes most malaria-related deaths, primarily in sub-Saharan Africa, whereas *P. vivax* (*Pv*), the most geographically widespread species, predominates in Latin America, Southeast Asia, and the Western Pacific^2^. Following mosquito transmission, *Plasmodium* sporozoites infect hepatocytes and undergo asexual replication (exo-erythrocytic or liver-stage schizogony), producing tens of thousands of merozoites released into the bloodstream^3^. Distinctively, a subset of *Pv* sporozoites form dormant liver stages known as hypnozoites, which can persist for weeks to years before activating and initiating schizogony, leading to relapse infections long after the primary episode^4,5^. In many endemic regions, relapses represent the predominant source of *Pv* blood-stage infections, complicating malaria control and elimination strategies^6–9^. Current relapse-prevention treatments using the drugs primaquine and tafenoquine, are contraindicated during pregnancy as well as for individuals with glucose-6-phosphate dehydrogenase deficiency (G6PD) or CYP2D6 polymorphisms, limiting their utility^9–11^. Consequently, there is a need for novel pharmacological interventions targeting latent hypnozoites.

For years, understanding hypnozoite biology was constrained by the lack of laboratory models to study *Pv* liver stages^7,12,13^. Over the past two decades, significant advances have emerged through improved infection systems, including in vivo humanized mouse models^7,14^ and in vitro hepatocyte platforms enabling *Pv* liver-stage infection and hypnozoite cultivation^15–17^. These models provided more insight into hypnozoite biology, including distinctive characteristics, frequency of occurrence, growth patterns, and activation processes^7,14,17^. Yet, the lack of facile *Pv* transgenesis has impeded insights into the molecular mechanisms governing hypnozoite formation, persistence, and activation. Recently, omics-based approaches have been applied to study gene expression in *Pv*^17–19^ and *Plasmodium cynomolgi*^20–22^ (a relapsing parasite of nonhuman primates) liver-stages revealed transcriptome differences between schizonts and hypnozoites, highlighting enriched hypnozoites transcripts for transcriptional regulators^17,19,20^, DNA and RNA-binding proteins^17–19,22^, factors involved in translational control^20^, and pathways governing microbial dormancy, genome integrity and ATP homeostasis^21^. However, the variation in gene expression patterns between different data sets complicates the identification of consistent, cross-species candidate genes that could regulate hypnozoite biology.

In this study, we compared *Pv*^17–19^ and *P. cynomolgi*^20–22^ liver-stage transcriptomes to identify molecular regulators potentially involved in hypnozoite formation, focusing on genes consistently enriched in hypnozoites. We hypothesized that hypnozoites must actively suppress the initiation of liver-stage schizogony to maintain quiescence and thus prioritized transcripts encoding transcriptional or translational regulators. This analysis identified two putative RNA-binding proteins expressed in *Pv* hypnozoites. Their functions were examined in *Plasmodium yoelii* (*Py*), a rodent malaria parasite that does not form hypnozoites. Both proteins were found to promote hypnozoite formation by repressing early liver-stage development and likely regulate gene expression by binding mRNAs via specific nucleotide motifs. Together, these findings implicate two RNA-binding proteins as key regulators of hypnozoite formation.

## Results

### Candidate gene identification and validation of differential expression in *P. vivax* liver stages

**Figure 1.**
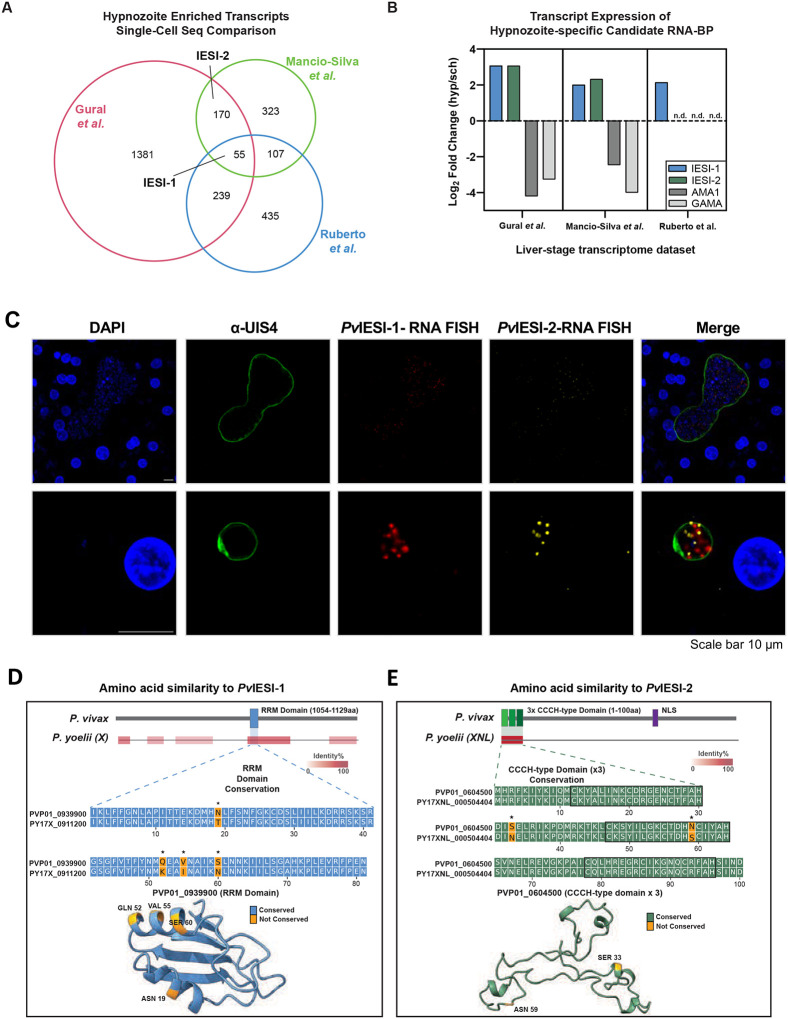
Identification and characterization of *Pv*IESI-1 and *Pv*IESI-2 as candidate regulators of hypnozoite development. **(A)** Comparison of published *P. vivax* hypnozoite transcriptomes. Differentially expressed transcripts from bulk RNA sequencing (Gural et. al, Mancio-silva et al. and scRNA-seq (Ruberto et al.) datasets were combined and cross-referenced to find overlapping genesets and the two candidate genes (IESI-1 and IESI-2) highlighted. **(B)** Specific examples of transcripts increased in liver stage schizogony (AMA1, GAMA) and IESI-1 and IESI-2 are shown with their relative expression in hypnozoites compared to liver schizonts in each liver-stage transcriptome dataset. **(C)** RNA-FISH analysis of *Pv*IESI-1 and *Pv*IESI-2 in HPP cells on day 8 post-sporozoite infection, showing transcriptional upregulation in hypnozoites compared to schizonts. **(D – E)** Schematic of the protein structures of *Pv*IESI-1 and *Pv*IESI-2 with identified domains and nuclear localization signal (NLS) identified in IESI-2. Regions of homology between *Pv* and *Py* IESI proteins are indicated by red bars, where intensity conveys % identity for each region. Below the schematics, the conservation of the IESI-proteins RRM and CCCH-type zinc finger domain sequences and structure between *Pv* and *Py* are highlighted with non-conserved residues indicated in orange and with * in the aligned sequences.

Because hypnozoites are thought to be actively quiescent, we sought to identify molecular factors that induce and maintain this cellular state by analyzing on hypnozoite-specific transcripts. We performed a comparative transcriptome analysis using three published *P. vivax* datasets^17–19^ (Figures 1A, S1A) and three *P. cynomolgi* datasets^20–22^, focusing on genes encoding for RNA-binding proteins that consistently expressed in hypnozoites relative to replicating liver schizonts. This analysis revealed two candidate genes, PVP01_0939900 and PVP01_0604500 (Figures 1A, 1B). The orthologs of these candidate genes were highly enriched in the bulk transcriptomes of hypnozoite in both *P. vivax*^17,18^ (Figure 1B) and *P. cynomolgi*^20,22^ (Figure S1B), while PVP01_0939900 was also enriched in a single cell transcriptome study of hypnozoites^19^ (Figure 1B). Based on phenotypic outcomes observed in this study, we have termed these proteins as Initiation of Exo-erythrocytic Schizogony Inhibited (IESI) proteins: *Pv*IESI-1 (PVP01_0939900) and *Pv*IESI-2 (PVP01_0604500).

To confirm the expression patterns of *Pv*IESI-1 and *Pv*IESI-2 transcripts, we performed RNA fluorescence in situ hybridization (RNA-FISH) on day 8 of *Pv* liver-stage development in primary human hepatocytes (Figure 1C). Indirect immunofluorescence assays (IFA) with the UIS4 antibody were used to confirm the identity of liver-stage parasites and visualize the parasitophorous vacuolar membrane (PVM) (Figure 1C). Probes targeting *Pv*IESI-1 (red) and *Pv*IESI-2 (yellow) transcripts were then used and showed predominant signals in small hypnozoite forms, with minimal detection in large schizonts (Figure 1C). These experimental findings show that *Pv*IESI-1 and *Pv*IESI-2 are transcribed specifically in hypnozoites, suggesting they might play a role in biological processes that are important to hypnozoite formation and persistence.

### *Pv*IESI-1 and *Pv*IESI-2 are predicted to be RNA-binding proteins

To explore the potental molecular roles of IESI-1 and IESI-2 in hypnozoite development, we analyzed their predicted protein structures. Both are annotated as putative RNA-binding proteins. *Pv*IESI-1 contains a single RRM motif (Figure 1D) classified within the RRM_1 sub-family (PF00076), though its function remain largely uncharacterized^23^. *Pv*IESI-2 belongs to the Zinc-finger (ZnF) class (Figure 1E) of proteins, comprising three ZnF CCCH-type domains (Figure 1E), and, like most *Plasmodium* CCCH-type proteins, its function is unpredicted due to the absence of orthologs in model species^23^.

Phylogenetic analysis showed that both IESI-1 and IESI-2 are conserved across the *Plasmodium* genus (Figures S1C, S1D). Their full sequences are conserved among Asian malaria species (Figures S1C, S1D), though *P. cynomolgi* IESI-1 orthologue is significantly shorter (1078 aa vs 1865 aa in *P. vivax*), sharing only the C-terminal region (Figure S1C). For both proteins, the RNA-binding domains are the most conserved regions among divergent *Plasmodium* species (Figures S1C, S1D), with only minor amino acid differences between human and rodent malaria parasites (Figures 1D, 1E). In fact, for IESI-2, the N-terminal zinc finger domains are the only regions of sequence conservation outside of the Asian *Plasmodium* parasite species, with sequence identity >70% for all *Plasmodium* orthologues (Figures S1C, S1D). This suggests that the RNA-binding function of these proteins is vital.

### Transgene expression of *Pv*IESI-1 and *Pv*IESI-2 in *P. yoelii*

Functional genetic analysis of *Pv* genes is difficult due to the lack of a facile transgenesis system. Thus, to investigate *Pv*IESI-1 and *Pv*IESI-2 in liver stages, we overexpressed them in the dispensable *Py* P230p locus using CRISPR/Cas9-assisted homologous recombination. The coding seqences of *Pv*IESI-1 and *Pv*IESI-2 were placed under control of *Py* UIS4 5’ and 3’ UTRs to restrict their respective expression to pre-erythrocytic stages (^*uis4*^*Pv*IESI-1 and ^*uis4*^*Pv*IESI-2). For localization studies, each protein was fused at the C-terminus with an mNeon tag (^*uis4*^*Pv*IESI-1^mNeon^ and ^*uis4*^*Pv*IESI-2^mNeon^, Figure S2A). Integration into clonal lines was confirmed by PCR and sequencing (Figures S2B, S2C). To assess potential effects of tagging, additional constructs expressing untagged *Pv*IESI-1/2 (Figure S3A) or mNeon alone (Figure S3B) were generated. PCR screening with recombination-specific primers confirmed successful integration events into the *Py*P230p locus (Figures S3C, S3D, S3E).

**Figure 2.**
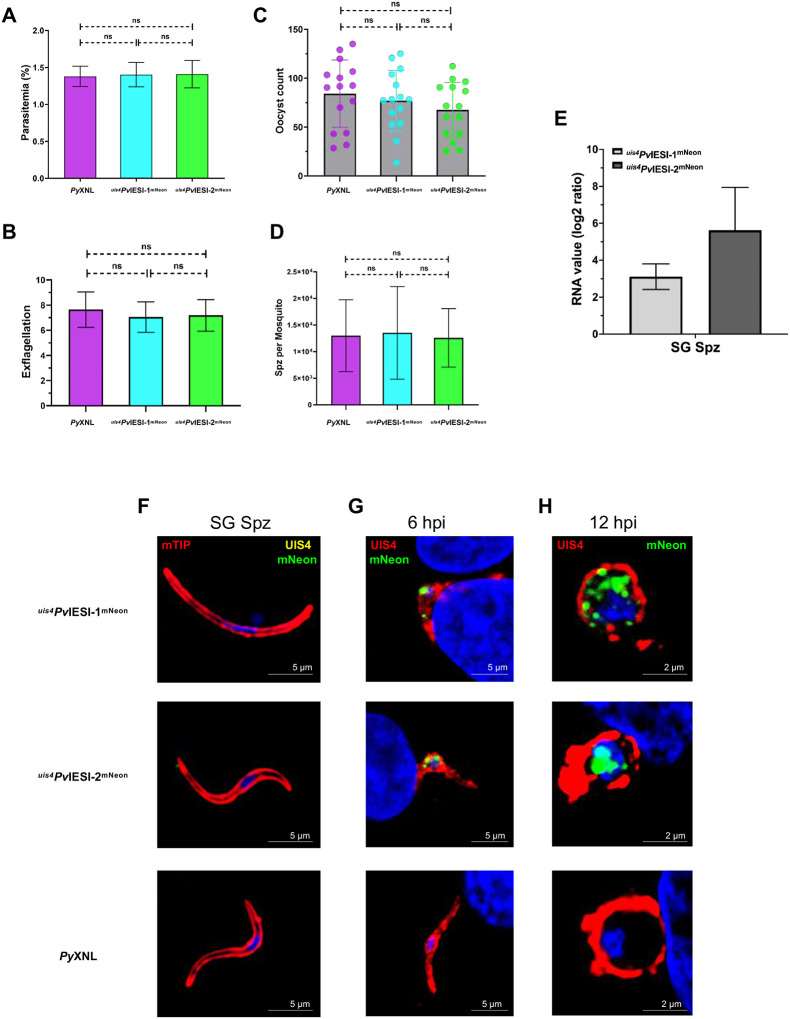
Overexpression of ^*uis4*^*Pv*IESI-1 and ^*uis4*^*Pv*IESI-2 does not impact parasite development in blood or mosquito stages. **(A)** Parasitemia levels were comparable between the wild-type *Py*XNL line and transgenic lines expressing ^*uis4*^*Pv*IESI-1^mNeon^ and ^*uis4*^*Pv*IESI-2^mNeon^. **(B)** Exflagellation centers showed no differences across 15 random fields at 7–10 minutes post-smear. **(C)** Oocyst counts on day 8 post-feeding and **(D)** sporozoite counts from salivary glands on days 14–15 post-feeding was unaffected by overexpression. **(E)** RT-qPCR analysis of ^*uis4*^*Pv*IESI-1^mNeon^ and ^*uis4*^*Pv*IESI-2^mNeon^ transcriptions, normalized to *Py*18S RNA. **(F)** Immunofluorescent assay (IFA) of salivary gland (SG) sporozoites (14–15 days post-infection) showing mTIP (red, indicated inner membrane complex), mNeon (green, *Pv*IESI-1^mNeon^/*Pv*IESI-2^mNeon^), UIS4 (yellow, parasitophorous vacuole membrane), and DAPI (nuclei). **(G – H)** IFA of ^*uis4*^*Pv*IESI-1^mNeon^ and ^*uis4*^*Pv*IESI-2^mNeon^ infected HepG2-CD81 cells at 6 hpi, and 12 hpi stained for mNeon (green) and UIS4 (red).

To compare the life cycles of the *P. yoelii* transgenic ^*uis4*^*Pv*IESI-1^mNeon^ and ^*uis4*^*Pv*IESI-2^mNeon^ lines with the wildtype (WT) *P. yoelii* XNL parental line (*Py*XNL), infected red blood cells containing each line were intraperitoneally injected into SW mice. Parasitemia and exflagellation (microgamete emergence) were quantified on days 3–4 post-infection. Giemsa-stained blood smears (parasitemia 1–2%; exflagellation 5–10 per field of view) indicated similar growth and gametocytogenesis rates between transgenic and WT parasites (Figures 2A, 2B). The transmissibility of gametocytes to *Anopheles stephensi* mosquitoes was assessed by dissecting infected midguts 8 days post blood feeding. ^*uis4*^*Pv*IESI-1^mNeon^ and ^*uis4*^*Pv*IESI-2^mNeon^ parasites did not show reduced oocyst numbers when compared to *Py*XNL (Figure 2C). Similarly, sporozoite counts in mosquito salivary glands at days 14–15 post blood feeding showed no negative impact on sporogony and salivary gland colonization in transgenic lines (Figure 2D).

Translation of UIS4 transcripts is well-documented to be repressed in sporozoites, enabling UIS4 protein synthesis only after transmission and host cell invasion^24–32^. We thus analyzed *Pv*IESI-1 and *Pv*IESI-2 expression in sporozoites of the ^*uis4*^*Pv*IESI-1^mNeon^ and ^*uis4*^*Pv*IESI-2^mNeon^ lines. We found that although the expression cassettes of both lines were transcriptionally active in salivary gland sporozoites (SG Spz) (Figure 2E), protein was undetectable when sporozoites were probed with mNeon antibodies (Figure 2F).

We next examined *Pv*IESI-1 and *Pv*IESI-2 protein expression in liver stages. Salivary gland sporozoites collected at days 14–15 post-blood feeding were used to infect HepG2-CD81 cells, and cultures were fixed at 6 and 12 hpi for staining with mNeon and UIS4 antibodies. IFA detected robust mNeon signal at 6 hpi, persisting at 12 hpi (Figure 2G, 2H), while WT *Py*XNL confirmed specificity. These data suggests that post-transcriptional silencing of the ^*uis4*^*Pv*IESI-1^mNeon^ and ^*uis4*^*Pv*IESI-2^mNeon^ expression is mediated by 5’ and/or 3’ UTR of UIS4, contrasting *P. berghei*, where UTRs are dispensable^33^. Early liver-stage quantification showed no significant difference between transgenic and WT *Py*XNL lines (Figure S4A), showing that the expression cassettes of transgenic lines did not impact sporozoite infectivity.

### Expression of *Pv*IESI-1 and *Pv*IESI-2 in *P. yoelii* perturbs liver stage to blood stage transition

**Figure 3.**
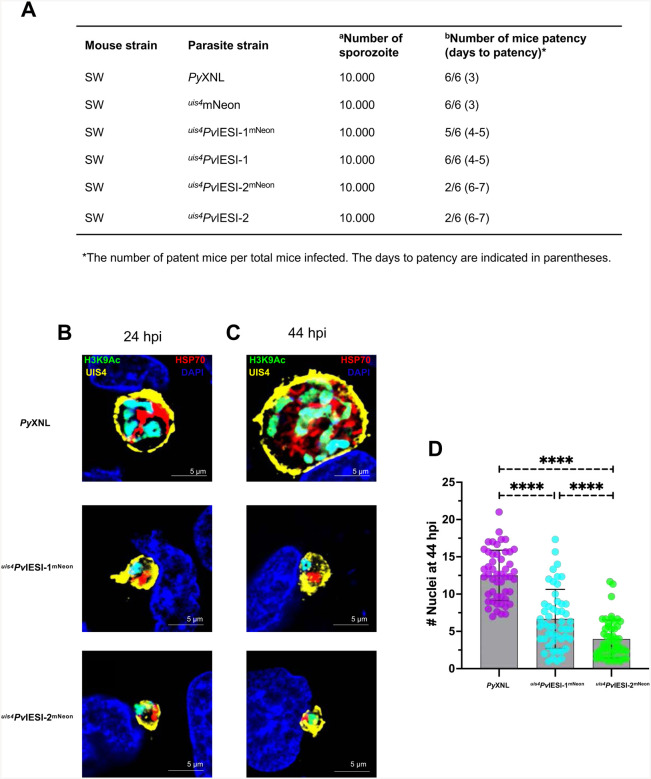
Overexpression of ^*uis4*^*Pv*IESI-1^mNeon^ and ^*uis4*^*Pv*IESI-2^mNeon^ perturbated liver-stage parasite development. **(A)** Table summarizes patency results: ^a^salivary gland sporozoites injected per mouse, and ^b^patent mice per total infected, with days to patency in parentheses. **(B–D)**Overexpression of ^*uis4*^*Pv*IESI-1^mNeon^ and ^*uis4*^*Pv*IESI-2^mNeon^ induced hypnozoite-like characteristics. Parasites were analyzed at 24 hpi **(B)** and 44 hpi **(C)** using H3K9Ac (green) indirectly marks nuclei, UIS4 (yellow) highlights PVM development, and HSP70 (red) identifies mitochondria. **(D)** the graph shows nuclei replication at 44 hpi across parasite lines, with error bars indicating standard deviation from three independent experiments.

We next performed analysis of the time to blood stage patency (prepatent period) by retro-orbital injection of 10.000 salivary gland sporozoites of each parasite line into Swiss Webster (SW) mice. Onset of blood stage parasitemia was monitored starting on day 3 post-infection (Figure 3A). Blood stage parasites were consistently detected in all six mice infected with WT *Py*XNL or *Py*XNL^mNeon^(^*uis4*^mNeon) sporozoites by day 3, indicating that pre-erythrocytic expression of mNeon did not cause any defects in the parasite. In contrast, prepatency was prolonged to days 4–5 in mice infected with the ^*uis4*^*Pv*IESI-1^mNeon^ and ^*uis4*^*Pv*IESI-1 lines, clearly indicating a perturbation of liver stage development. Expression of *Pv*IESI-2 even more dramatically impaired liver-stage development. In the ^*uis4*^*Pv*IESI-2^mNeon^ line, only 2 out of 6 mice developed blood-stage parasitemia, which was detectable on days 6–7 post-infection. Similarly, in the ^*uis4*^*Pv*IESI-2 line, blood stage parasitemia was also observed in only 2 of 6 mice. These results show that *Pv*IESI-1 and *Pv*IESI-2 expression in *Py* liver stages severly impacts liver-stage development and formation of infectious exo-erythrocytic merozoites.

### *Pv*IESI-1 and *Pv*IESI-2 inhibit exo-erythrocytic schizogony

Hypnozoites are notably distinguished from replicating liver stage schizonts by their small size^8^, minimal DNA replication, and underdeveloped organelles^7,17^. To assess liver stage phenotypes of *Pv*IESI-1 or *Pv*IESI-2, IFAs were performed on ^*uis4*^*Pv*IESI-1^mNeon^ and ^*uis4*^*Pv*IESI-2^mNeon^ lines and compared to WT *Py*XNL line at 24 hours hpi (Figure 3B) and 44 hpi (Figure 3C) in HepG2-CD81 cells. Liver stages showed strong circumferential UIS4 staining of parasitophorous vacuole membrane (PVM). Strikingly, transgenic parasites remained small at both time points, whereas WT parasites exhibited robust growth. An antibody specific for histone acetylation (H3K9Ac, green) was used to indirectly assess DNA replication. Remarkably, the small ^*uis4*^*Pv*IESI-1^mNeon^ and ^*uis4*^*Pv*IESI-2^mNeon^ liver-stage parasites consistently displayed a single acetylated histone-positive structure, clearly indicating minimal genome replication and segregation at 24 hpi (Figure 3B) and 44 hpi (Figure 3C), while WT parasites progressively accumulated multiple structures. DAPI/H3K9Ac co-staining quantified DNA center at 44 hpi (WT averaged 12.5, ^*uis4*^*Pv*IESI-1^mNeon^ 6.7 and ^*uis4*^*Pv*IESI-2^mNeon^ 4) (Figure 3D), demonstrating that *Pv*IESI-1/2 expression suppresses DNA replication and liver-stage growth.

As liver-stage schizogony advanced in the WT *Py*XNL line exhibited progressive growth and mitochondrial branching. Conversely, only a single mitochondrial structure was consistently observed in a subset of parasites in the ^*uis4*^*Pv*IESI-1^mNeon^ and ^*uis4*^*Pv*IESI-2 ^mNeon^ lines (Figures 3B, 3C), strongly suggesting a significant suppression of organelle expansion and development.

**Figure 4.**
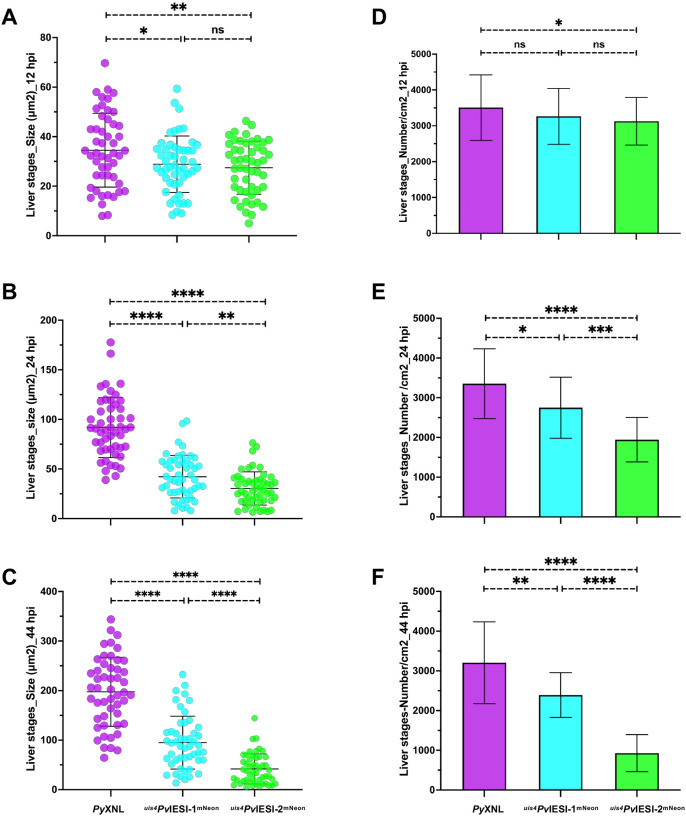
*Pv*IESI-1 and *Pv*IESI-2 inhibit exo-erythrocytic schizogony and reduce parasite survival. **(A–C)** Liver-stage parasite size over time showing reduced growth in parasites expressing *Pv*IESI-1 or *Pv*IESI-2. **(D–F)** Parasite numbers during the developmental time course, indicating a progressive decline, with a more pronounced defect observed upon *Pv*IESI-2 expression. Errors bars represent standard deviation (SD) from four independent experiments.

We quantitatively analyzed liver-stage growth of ^*uis4*^*Pv*IESI-1^mNeon^ and ^*uis4*^*Pv*IESI-2^mNeon^ at different time points in vitro. IFAs and size measurements revealed that expression of *Pv*IESI-1 and *Pv*IESI-2 resulted in stunted liver stage development. At 12 hpi, the size differences between the WT *Py*XNL and the transgenic lines were minimal (Figure 4A). However, by 24 hpi, a significant reduction in liver stage size was observed for both transgenic lines when compared to WT *Py*XNL (Figure 4B). At 44 hpi, most WT *Py*XNL parasites had grown to well developed liver-stage schizonts. In stark contrast, ^*uis4*^*Pv*IESI-1^mNeon^ and ^*uis4*^*Pv*IESI-2^mNeon^ liver stages exhibited continued growth arrest and maintained a population of small parasites (<5 μm) (Figure 4C). Interestingly, this lack of growth was more pronounced for the ^uis4^*Pv*IESI-2^mNeon^ liver stages (Figures 4B, 4C). Similar phenotypes were also observed with ^*uis4*^*Pv*IESI-1 and ^*uis4*^*Pv*IESI-2 liver-stage lines lacking mNeon (Figure S4B), confirming that growth arrest is specifically attributable to *Pv*IESI-1/2 expression.

We next quantified liver-stage parasite numbers in transgenic lines compared to WT *Py*XNL over time. At 12 hpi, comparative liver stage parasite numbers were similar for transgenic and WT lines (Figure 4D). By 24 and 44 hpi however, the transgenic lines showed a decline in liver stage numbers compared to WT *Py*XNL, with a more pronounced declines observed for ^*uis4*^*Pv*IESI-2^mNeon^ liver stages (Figure 4E, 4F).

### *Pv*IESI-1 and *Pv*IESI-2 localize to liver stage cytoplasmic stress granules

**Figure 5.**
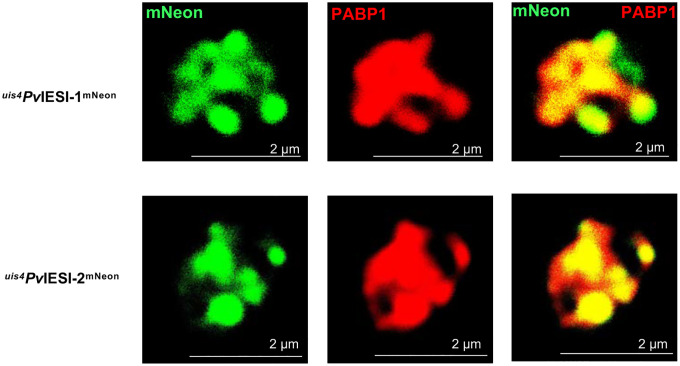
*Pv*IESI-1 and *Pv*IESI-2 localize to liver-stage cytoplasmic stress granules, as indicated by co-localization with PABP1.

*Pv*IESI-1 and *Pv*IESI-2 harbor RNA-binding domains and their individual expression in *P. yoelii* liver stages inhibits liver-stage schizogony. One mechanism by which they might excert this effect is translational control via regulation of RNA stability and translational repression, which in *Plasmodium* ssp occurs in stress granules^34–37^. To examine the localization of *Pv*IESI-1 and *Pv*IESI-2, we performed IFAs on ^*uis4*^*Pv*IESI-1^mNeon^ and ^*uis4*^*Pv*IESI-2^mNeon^ liver-stage infection in HepG2-CD81 cells. The mNeon signal indicated that *Pv*IESI-1 and *Pv*IESI-2 predominantly localize to granular structures (Figures 2G, 2H; S4C, S4D). We next compared *Pv*IESI-1 and *Pv*IESI-2 localization to that of poly (A)-binding protein 1 (PABP1), a protein known to associate with translational repression complexes, including those found in stress granules in *Plasmodium* spp^38,39^. As PABP1 binds to the poly (A) tail of mRNAs, its distribution highlights regions where mRNA is actively localized^40,41^. We observed strong co-localization between the *Pv*IESI-1^mNeon^ and *Pv*IESI-2^mNeon^ signals with PABP1 (Figure 5), strongly suggesting their localization to stress granules.

### IESI-1 and IESI-2 bind sequence-specific RNA

**Figure 6.**
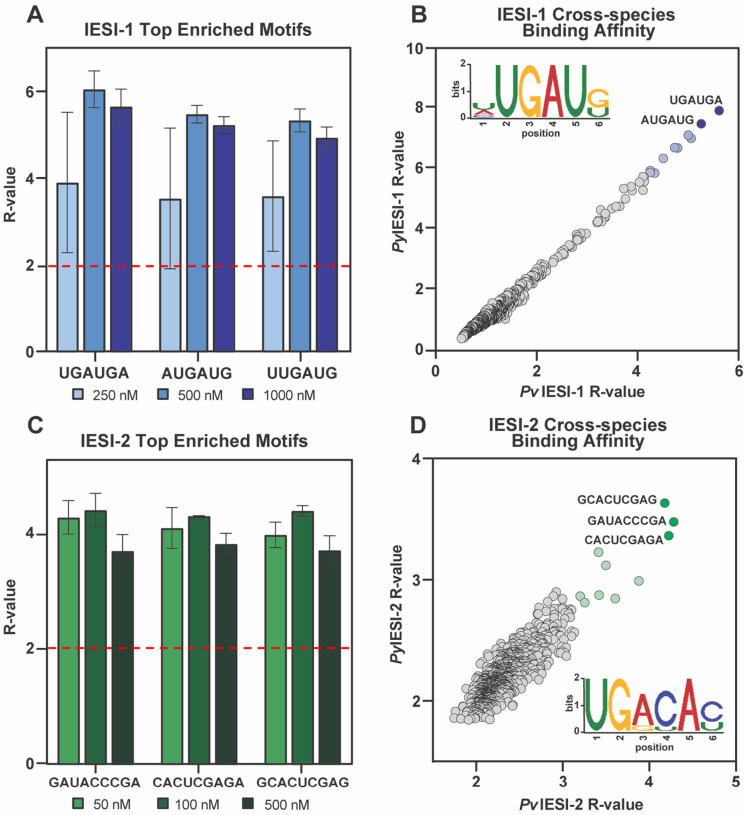
Determining the *in vitro* RNA binding specificity of IESI-1 and 2. Results from RNA-bind-n-seq shows the top motifs enriched above occurrences of all possible 6-mers as R-values with significance threshold indicated with dashed red line for *Pv*IESI-1 **(A)** and *Pv*IESI-2 **(C)** respectively. Correlation between the average enrichment (R-values) obtained for motifs in the *P. yoelii* and *P. vivax* IESI-1 **(B)** and IESI-2 **(D)** experiments. Representative sequence logo of 10 top motifs enriched in RNA-bind-n-seq was generated in seqlogo in R.

Recognizing the importance of both *Pv*IESI-1 and *Pv*IESI-2 to liver-stage development, we investigated their potential role as mRNA interactors based on predicted RNA-binding domains. Since functional study in *P. vivax* liver-stages are challenging, we opted for characterizing the function of *Pv*IESI-1 and *Pv*IESI-2 as recombinantly expressed proteins *in vitro* using RNA-Bind-n-Seq (RBNS)^42^. This approach identifies RNA sequences preferentially bound by the respective RNA-binding domains of either IESI-1 or IESI-2. The recombinant domains were incubated with 40-mer random RNA library with titrating concentrations of either the recombinant RRM domain from IESI-1 or the 3x CCCH-type zinc finger domain from IESI-2. The protein-bound RNAs are isolated and identified by next-generation sequencing. The top enriched sequences were determined relative to the frequencies in a sequenced input library control^42^ across two replicates for each protein domain of interest (Figure 6).

Across all tested concentrations, the *Pv*IESI-1 RRM showed strongest enrichment for a 6-mer RNA sequence 5’-UGAUGA-3’ (R-value average = 5.2±0.6), with other top hits containing the 5’-UGA-3’ core (Figure 6A). To assess conservation, RRM orthologs from *P. falciparum* (PF3D7_1139100) and *P. yoelii* (PY17X_0911200) (Figures S1C) were analyzed. Both orthologs displayed identical motif enrichment, with 5’-UGAUGA-3’ as the top sequence (Figures 6A, S5A, S5B), and top results were highly correlated across species (Figures 6B, S5C). These findings demonstrate that the *Pv*IESI-1 RRM selectively binds RNA sequences containing the conserved 5’-UGA-3’ core motif.

Similarly, we then used RBNS to determine the binding specificity of *Pv*IESI-2 3xCCCH-type zinc-finger domains. The highest R-value results were obtained for 9-mer sequences, with 5’-GAUACCCGA-3’ enriched (average R-value = 4.3±0.2) (Figure 6C). Analysis of top bound sequences revealed a conserved 5’-UGAYAYYYG-3’ consensus motif (preferring cytosines at Y positions) across species (*P. yoelii*: Py17XNL_000504404 and *P. falciparum*: PF3D7_1019300), with 5’ portion, 5’-UGAYAC-3’, showing the strongest enrichment (Figures 6D, S5D-S5F).

Pyrimidines at Y positions were predominantly cytidine, and a preceding uracil defined the most conserved 6-mer, 5’-UGACAC-3’ (Figure 6D). Since each CCCH-type zinc-finger domain typically binds a triplet nucleotide sequence with varying affinities between domains^43,44^, 5’-UGACAC-3’ motif likely represents the preferred binding site, allowing two zinc fingers to interact simultaneously, rather than requiring the full 9-mer sequence. Together, these data indicate that *Pv*IESI-1 and *Pv*IESI-2 are RNA-binding proteins with distinct sequence preferences.

### *Pv*IESI-1 and *Pv*IESI-2 binding sequences are enriched within the *P. vivax* liver stage transcriptome

**Figure 7.**
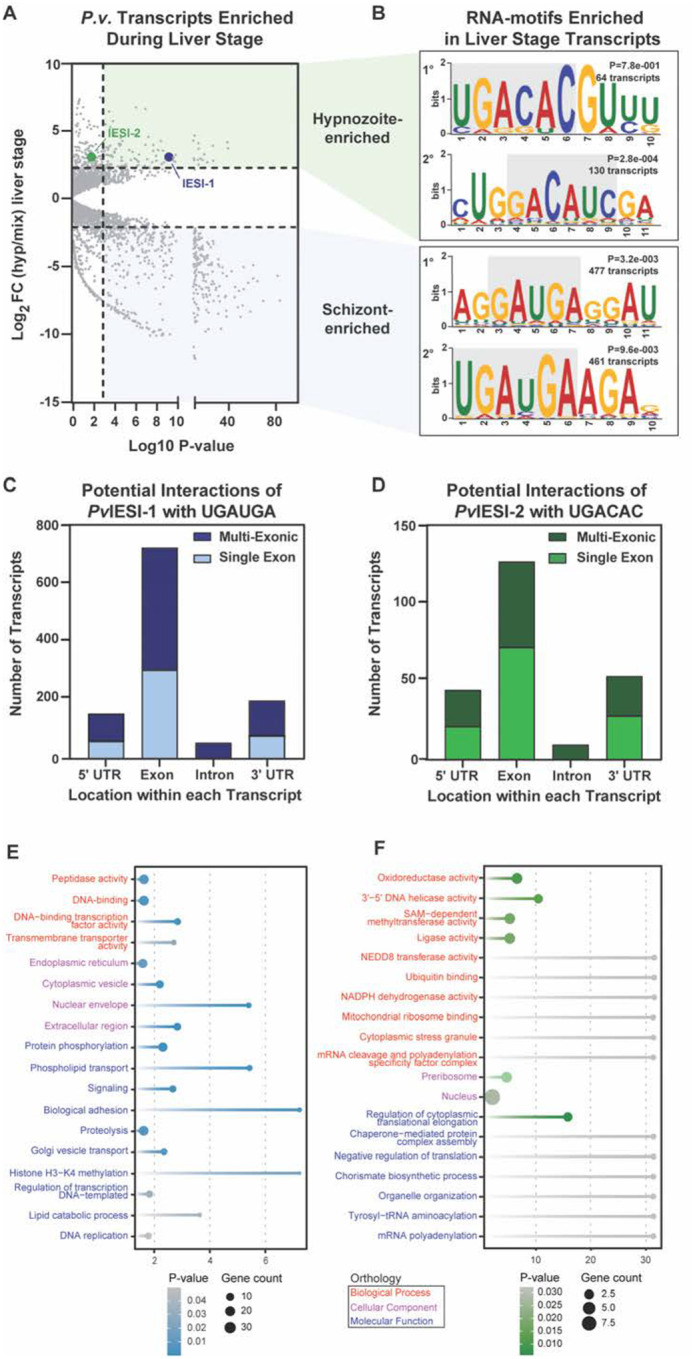
The IESI-1 and 2 preferred binding sequences are relevant to *P. vivax* liver stage development. **(A)** The transcripts identified as differentially expressed in Gural et al. are visualized as a volcano plot, and **(B)** enriched motifs for each set were obtained using STREME with the *P. vivax* transcriptome as background **(C, D)** The occurrences and locations of the IESI-1 and 2 binding sequences in the *P. vivax* transcriptome are indicated in stacked bar graphs. **(E)** Transcripts enriched in exoerythrocytic schizont stages containing the IESI-1 preferred motif (UGAUGA) and **(F)** hypnozoite transcripts containing the IESI-2 preferred motif were searched for enriched gene ontology terms (GO) using PlasmoDB (www.plasmodb.org) with *P*<0.05.

We next interrogated the *P. vivax* hypnozoite and liver-schizont transcriptomes to identify potential binding sequences for *Pv*IESI-1 and *Pv*IESI-2, aiming to uncover their regulatory roles during the liver-stages development. Using an agnostic motif search across all liver-stages-enriched transcripts, we assessed whether IESI-1 and IESI-2 preferred binding motifs are relevant to liver-stage *P. vivax* development. Differentially expressed transcripts from Gural *et al*.^17^ (Figure 7A; Log_2_ Fold Change >1, *P*<0.05) was examined for motif enrichment linked to either hypnozoite or schizont stages. Multiple motifs were identified as enriched above background (*P*<0.05, Supplementary File Y). In schizonts, full (5’-UGAUGA-3’), *P*=9.6e-003) and partial (5’-GAUGA-3’, *P=*3.2e-003) IESI-1 motifs appeared in 45% and 44% of transcripts, respectively (Figure 7B). In hypnozoite, we also identified both the full (UGACAC, *P=* 7.8e-0.001) and partial (GACAUC*, P=2.8e-004*) IESI-2 motifs were enriched in 20% and 10% of transcripts (Figure 7B).

When we probed all liver-schizont enriched transcripts for the IESI-1 motif (Figure 7A; 1,059 transcripts), 79% contained at least one instance of the sequence. Because motif localization can functional significance, we examined the distribution of 5’-UGAUGA-3’ motifs, which was significantly skewed towards exons, mostly in multi-exonic transcripts (60%, Figure 7C), suggesting a role in transcript splicing. The IESI-2 motif (5’-UGACAC-3’) appeared in 33% of hypnozoite-enriched transcripts, primarily within coding regions (Figure 7D). Unlike IESI-1, IESI-2 motifs was less exon-biased (Figure 7A; 653 transcripts), and more likely associated with introns or UTRs (Figure 7D), indicating possible translational regulatory functions.

We then performed a GO enrichment analysis on the transcripts containing each corresponding motif to determine potential functional categories co-regulated by *Pv*IESI-1 and *Pv*IESI-2. Transcripts containing IESI-1 motifs were strongly enriched for phosphate metabolism, particularly protein phosphorylation (GO:0006468) and phospholipid transport (GO:0015914) (Figure 7E). Notably, 25 kinases and 6 phospholipid transporters contained the IESI-1 motif, suggesting IESI-1 may coordinate cellular energy management. These transcripts were also enriched for pathways essential to schizont development, including DNA replication (GO:0006260) and signaling (GO:0023052) (Figure 7E). Among these are the most highly upregulated exported proteins identified in schizonts by Gural *et al*.^17^ (PVP01_0533700, PVP01_1402700, PVP01_0503800). Interestingly, IESI-1 motifs were also present in transcriptional regulation (GO:0006355), including ten ApiAP2 transcription factors such as AP2-I and AP2-LT (Figure 7E). This pattern suggests a regulatory hierarchy: IESI-1 may act as a master switch, potentially suppressing schizont-promoting factors to block schizogony.

GO enrichment analysis of hypnozoite transcripts containing IESI-2 motifs revealed potential links to dormancy-associated cellular machinery. These potential target transcripts orchestrate both RNA metabolism – including cytoplasmic translation elongation (GO:1900247), mRNA polyadenylation (GO:0006378), and stress granule formation (GO:0010494) – and protein metabolism via ubiquitin binding (GO:0043130) and NEDD8 transferase activity (GO:0019788) (Figure 7F). Enrichment for negative regulation of translation (GO:0017148), include a G-coupled protein receptor (PVP01_1008400) and the known translational repressor PUF1 (PVP01_1015200), which carries the IESI-2 motif (Supplementary File Y). Additionally, pre-ribosome components (GO:0030684), such as BRX1 homolog (PVP01_0215100) and multiple RNA helicases (PVP011403600, PVP01_1408200) are enriched. These findings implicate *Pv*IESI-2 as a key regulator of translational control, fine-tuning the protein synthesis machinery to promote and sustain hypnozoite dormancy.

## Discussion

When *P. vivax* sporozoites infect hepatocytes, they develop into trophozoites that either initiate immediate exo-erythrocytic schizogony, leading to primary blood stage infection or form dormant hypnozoites, which persist in the liver and can activate after weeks or months to undergo secondary schizogony, which produces exo-erythrocytic merozoites long after primary infection and causes relapses^4,5^. Establishing this non-replicative state requires pausing cell cycle progression and preventing schizogony. We hypothesized that hypnozoites actively suppress replication through translational repression^9^. Transcriptomic analyses of *P. vivax*^17–19^ and its model *P. cynomolgi*^20–22^ revealed distinct expression profiles between hypnozoites and schizonts, yet cross-study variability has hindered the identification of conserved regulators. To resolve this, we performed a comparative analysis and identified two hypnozoite-enriched RNA-binding protein genes, IESI-1 and IESI-2, confirmed by gene-specifc RNA-FISH.

The study of hypnozoite formation in *P. vivax* is limited by the absence of a continuous blood-stage culture and a facile transgenesis system, preventing generation of transgenic gametocytes, mosquito stages, and sporozoites^8,45^. Here, we used the *P. yoelii* model and its robust genetic tools to investigate *P. vivax* protein function. As *P. yoelii* lacks dormant liver stages, we hypothesized that expressing hypnozoite-associated genes could reveal latency mechanisms. Using UIS4 regulatory elements, *Pv*IESI-1 and *Pv*IESI-2 expression initiated post-hepatocyte infection, without affecting blood-stage replication, gametocytogenesis, mosquito development, or sporozoite infectivity.

Hypnozoites are defined by their small cell size^8^, absence of DNA replication, and lack of organelle expansion such as the endoplasmic reticulum, mitochondria, and apicoplast^7,17^, which clearly distinguishes them from replicating liver-stage schizonts. Overexpression of *Pv*IESI-1 or *Pv*IESI-2 in *P. yoelii* liver-stages recapitulated these features, producing static, uninuclear cells with underdeveloped organelles. Both transgenes delayed liver-to-blood stage transition compared to wild-type *PyXNL*, with *PvIESI-2* showing a stronger inhibitory effect. This establishes *Pv*IESI-1 and *Pv*IESI-2 as potential regulators of developmental arrest and suggests they could be critical molecular switches controlling hypnozoite formation. Interestingly, a significant population of parasites with larger liver-stage sizes for ^*uis4*^*Pv*IESI-1^mNeon^ and ^*uis4*^*Pv*IESI-2^mNeon^ parasites showed a diminished mNeon signals (Figures S4C, S4D), indicating declining *Pv*IESI expression. However, whether this declining expression is mechanistically linked to parasites entering schizogony cannot be ascertained from these observations.

Translational repression mediated by cytoplasmic granules is a well-established regulatory mechanism in *Plasmodium* transmission stages. In gametocytes, maternal mRNAs are sequestered within DOZI- and CITH-containing messenger ribonucleoprotein (mRNP) complexes to ensure timely expression after fertilization^36,46,47^. Similarly, in sporozoites, RNA-binding proteins such as Puf2 and ALBA family members suppress translation to maintain infectivity until host invasion^35,48^. These stress granule–like assemblies underscore an evolutionarily conserved post-transcriptional control that ensures precise temporal protein expression just-in-time during host transitions. *Pv*IESI-1 and *Pv*IESI-2 were continusly expressed between 6–44 hours post-sporozoite infection and primarily localized to cytoplasmic granules. Their co-localization with the stress granule-associated protein PABP1 in *Plasmodium* spp^38,39^, indicates a role in RNA regulatory complexes, potentially involved in translational repression. These features align with their identity as RNA-binding proteins and point to a possible function in modulating mRNA fate during liver-stage development. By influencing mRNA stability or translation, *Pv*IESI-1 and *P*vIESI-2 might contribute to hypnozoite formation, highlighting them as potential targets for disrupting *P. vivax* persistence. However, since these expression and localization profiles originate from an artificial overexpression system, it will be important to further investigate their endogenous expression and localization using *P. vivax* hepatocyte infection models.

Given their annotation as RNA-binding proteins and their strong effects on liver-stage development, we experimentally verified *Pv*IESI-1 and *Pv*IESI-2 as bona fide RNA-binding proteins. Using RNA Bind-N-Seq (RBNS), we identified distinct sequence motifs bound by the RNA-binding domains of IESI-1 and IESI-2 orthologues across three *Plasmodium* species. Although this *in vitro* approach cannot fully capture the spectrum of IESI transcripts-bound as using *in vivo* approach like cross-linking immunoprecipitation and sequencing (CLIP-seq), it directly confirms specific RNA-binding activity to conserved motifs^42^ in systems such as *P. vivax* where generating samples for *in vivo* approaches may be untenable. The high conservation and sequence specificity of the IESI RNA-binding domains among *P. vivax*, *P. yoelii*, and *P. falciparum* underscore their essential molecular function.

The preferred binding motif of IESI-1 appears across a broader mRNA target repertoire in liver-stage parasites, suggesting modulatory role in stress adaptation or developmental plasticity rather than direct dormancy enforcement. *Pv*IESI-1, annotated as a paralog of CELF1 (PVP01_1112500) and showing the phylogenetic similarity to splicing factor 3B, subunit 4 (PTHR15241:SF62, E-value= 1e-36), may participate in RNA processing. Its *P.cynomolgi* ortholog (PCYB_094480, 74% identity) is predicted to positively regulate mRNA splicing via the spliceosome (GO:0048026). Consistently, the human CELF1 binding motif (5’-UGU-3’)^42^ resembles to one to two instances of the 5’-UGA-3’ sequence enriched in IESI-1 targets, supporting a posible role for IESI-1 in RNA splicing or processing. The IESI-1 motif is enriched in multi-exonic, highly expressed schizont-stage transcripts associated with DNA metabolism, helicase activity, and transcriptional regulation. The precise nature of regulation by *Pv*IESI-1 remains uncertain, as CELF family splicing factors can function as either activators or repressors depending on ther interaction partners *in vivo*^49^. Consistently, *Pv*IESI-1 overexpression only modestly affected liver-stage development, possibly due to altered protein interactions in *P. yoelii* compared with those in *P. vivax* hypnozoites.

*Pv*IESI-2 is annotated as a paralog of PVP01_0725700, the syntenic ortholog of gametocyte development protein 1 (GD1) in *P. berghei*, a gene essential for the production of gametocytes in rodent malaria species^50^. Although the function of both paralogs is still unexplored in *P. vivax*, the *Pv*IESI-2 binding motif is enriched in hypnozoite-associated transcripts, and its perturbation causes a strong schizogony inhibition. These findings suggests that *Pv*IESI-2 may function as a key effector of dormancy, possibly through the activation of hypnozoite-related transcripts, while *Pv*IESI-1 provides a broader regulatory framework that supports stage transitions. Together, *Pv*IESI-1 and *Pv*IESI-2 might mediate post-transcriptional programs that coordinate the balance between dormancy and replication in *P. vivax* liver-stage development. Although *in vitro* RBNS identified specific RNA motifs bound by *Pv*IESI-1 and *Pv*IESI-2, in vivo validation through CLIP (UV cross-linking and immunoprecipitation) or RIP (RNA immunoprecipitation) will be helpful to identify their direct or indirect mRNA aasociations. While such experiments remain challenging in *P.vivax*, they are feasible in genetically tractable models such as *P.falciparum* and *P.yoelii*, whose conserved IESI homologs share nearly identical RNA-binding motifs.

The RNA-binding domains of IESI-1 and IESI-2 are highly conserved across *Plasmodium* species, suggesting a shared molecular function in RNA recognition. However, despite this conservation, the *P.vivax* proteins uniquely suppress schizogony during liver-stage development, whereas the *P. falciparum* and *P. yoelii* orthologs do not exhibit this phenotype under natural conditions. This divergence in functional outcome implies that regions outside the conserved RNA-binding domains might play a crucial role in determining species-specific regulatory functions. Indeed, sequence comparisions reaveal substantial divergence in the non-RNA-binidng regions among *Plasmodium* species, which may modulate protein-protein interactions, subcellular localization, or post-translational modifications, ultimately shaping their roles in liver-stage development and dormancy regulation. In addition, differences in species-specific expression profiles might contribute to their phenotypic effects in different parasite life cycle stages.

Our data reveal important functions of *Pv*IESI-1 and *Pv*IESI-2 using the *P.yoelii* model. However the formation and persistence of hypnozoite in *P.vivax* are likely more complex than regulation by *Pv*IESI-1 or *Pv*IESI-2 alone. Many more factors must be involved, particularly those that enable hypnozoites to survive within hepatocytes. The fact that ^*uis4*^*Pv*IESI-1^mNeon^ or ^*uis4*^*Pv*IESI-2^mNeon^ parasites do not survive very well in *P.yoelii* model suggests that this species lacks key survival mechanisms of growth and replication-quiescent *P.vivax* hypnozoites.

**Figure 8.**
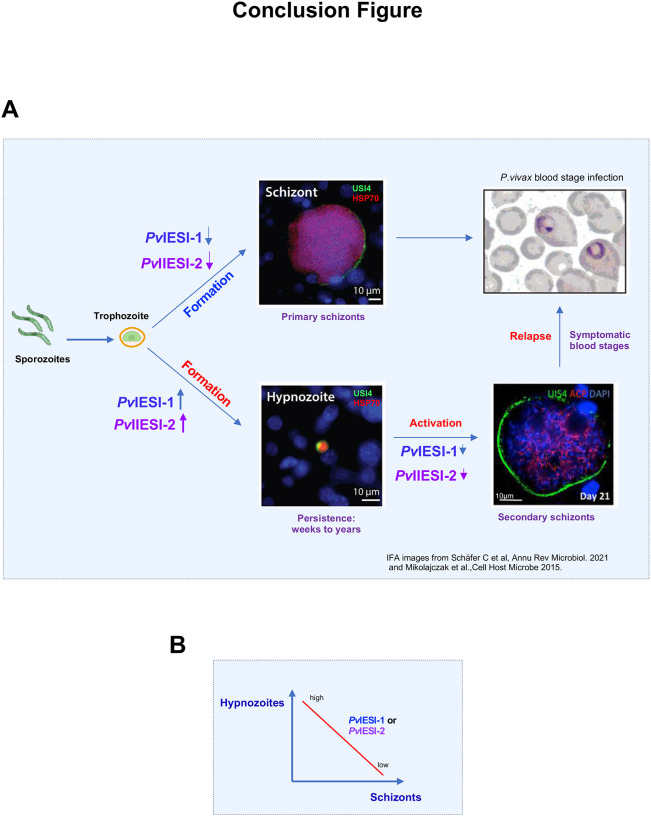
Proposed model for *Pv*IESI-1 and *Pv*IESI-2 – mediated regulation of liver-stage fate. **(A)** Schematic model illustrating how the formation of either replicating schizonts or dormant hypnozoites may depend on the expression levels of *Pv*IESI-1 and *Pv*IESI-2. **(B)** Simplified concept depicting that parasite growth or dormancy is determined by the relative abundance of *Pv*IESI-1 and *Pv*IESI-2.

In summary, this study presents a direct functional analysis of candidate hypnozoite regulators of *P. vivax*. Our findings suggest that both *Pv*IESI-1 and *Pv*IESI-2 contribute to the control of hypnozoite dormancy via post-transcriptional regulation of transcripts required for schizogony. Here, we propose a model in which elevated expression and RNA-binding activity of *Pv*IESI-1 and *Pv*IESI-2 promote hypnozoite formation by repressing transcripts required for schizogony. Conversely, when expression of these proteins is low, repression is relieved, allowing parasites to exit dormancy and enter schizogony. In established hypnozoites, a decline in *Pv*IESI-1 and *Pv*IESI-2 expression levels may trigger activation, initiating secondary schizogony and subsequent blood-stage relapse, as illustrated in Figure 8.

## EXPERIMENTAL PROCEDURES

### Experimental animals and parasite production

Seven-to eight-week-old female Swiss Webster (SW) mice purchasing from Envigo were used for the maintenance of *Py* life cycle and generation of transgenic parasites. The human hepatocyte (HepG2-CD81) cells were used to infect with sporozoite of the *Py*XNL and transgenic strains.

### Creation of *P. yoelii* expressing *Pv*IESI-1 ORF *and Pv*IESI-2 ORF

The vector containing the *p*UIS4 promoter, and 5’and 3’flanking regions of *Py*P230P was previously generated by a former lab member using the pYC vector backbone^51^. Subsequently, the mNeon sequence was ligated into the vector by using the KpnI/NheI restriction enzymes to create the pUIS4-mNeon vector (Figure S3B). Finally, the codon optimized *Pv*IESI-1 ORF and *Pv*IESI-2 ORF (obtained from GENEWIZ and GenScript, respectively) were ligated into the vectors (containing and not containing mNeon) at restriction sites PmeI/KpnI to creating four complete pUIS4-*Pv*IESI-1^mNeon^ and pUIS4-*Pv*IESI-2^mNoen^ (Figure S2A), pUIS4-*Pv*IESI-1 and *Pv*IESI-2 vectors (Figure S3A). These vectors were transfected into the blood stage schizonts of *Py*XNL to create the overexpressed *Pv*IESI-1 and *Pv*IESI-2 strains using CRISPR/Cas9 methodology. After transfection and retro-orbital injection into SW mice, pyrimethamine (7g/ml) was used for the positive selection. The clonal recombinant transgenic parasites were isolated by the limiting dilution, as described elsewhere^52,53^. All oligonucleotide primers used for the generation and genomic screening of ^*uis4*^*Pv*IESI-1^mNeon^, ^*uis4*^*Pv*IESI-2^mNeon^, ^*uis4*^*Pv*IESI-1, ^*uis4*^*Pv*IESI-2 and ^*uis4*^mNeon lines are listed in Table S1.

### In vivo and in vitro sporozoites infection

The *A. stephensi* mosquito’s salivary gland infected with 6 parasite lines (*Py*XNL, ^*uis4*^*Pv*IESI-1^mNeon^, ^*uis4*^*Pv*IESI-2^mNeon^, ^*uis4*^*Pv*IESI-1, ^*uis4*^*Pv*IESI-2 and ^*uis4*^mNeon), sprozoites were dissected between 14–16 days after the primary infectious blood meal. The alive-inactive sporozoites in ice-Schneider buffer were retro-orbital injected into SW mice (10.000 Spz/mouse, for patent examination). To set up the invitro infection, 5×10^4^ - 7.5×10^4^ Spz were seeded into the confluent HepG2-CD81 cells, and sampled at 6 hours, 12 hours, 24 hours, and 44 hours’ time points.

### RNA-FISH assay

RNA fluorescence in situ hybridization (RNA-FISH) combined with immunofluorescence assay (IFA) was performed on *Plasmodium vivax* liver-stage schizonts and hypnozoites derived from infected primary human hepatocytes (BioIVT, batch #BGW) at day 8 post-infection. The RNAscope Multiplex Fluorescent Assay v2 (Advanced Cell Diagnostics) was carried out according to the manufacturer’s instructions. Cells were treated with protease Solution 3 (1:10 dilution) for 20 minutes at room temperature, followed by overnight incubation with mouse monoclonal anti-*Pv*UIS4 antibody (1:1000 dilution)^54^, and fixed in neutral-buffered formalin for 10 minutes.

Probe hybridization was performed for 2 hours at 40 °C using probes targeting *P. vivax* transcripts PVP01_0939900 (20ZZ probe, regions 2–1056) and PVP01_0604500 (20ZZ probe, regions 2702–3701). Signal amplification was achieved using tyramide signal amplification (TSA) with TSA Vivid fluorophores, following the manufacturer’s protocol. Cells were counterstained with Alexa Fluor 488-conjugated secondary antibody and DAPI. Imaging was performed using the Stellaris 8 confocal microscope (Leica Microsystems), with image processing via Leica Lightning and analysis conducted in Fiji (ImageJ).

### qRT-PCR

1 × 10^6^ to 2 × 10^6^ SG Spz for each parasite strain was used to extract the total RNA, using Qiagen miRNeasy mini kit (cat. no. 217004). QuantiTect-Qiagen Reverse transcription kit (Cat. No. 205311) was used to reverse RNA to cDNA. qRT-PCR was performed using SYBR Green Master Mix (cat. no. B21202) and Reference Dye 2 on a Real-time PCR instrument (QuantStudio 5). Specific primers for the target genes, and *Py*18S RNA gene were designed using PrimerQuest^™^ Tool (https://www.idtdna.com/PrimerQuest/Home/Index). The primer sequences are detailed in Table S1. Relative gene expression was calculated using the 2^−ΔΔCt method, with normalization to the control *Py*18S RNA gene. All reactions were performed in triplicate.

### Indirect immunofluorescence assay (IFA)

IFA was performed as previously described^55^. Briefly, either salivary gland sporozoites (SG Spz) or SG-infected HepG2 + CD81 cells (at 6, 12, 24, or 44 hpi) were fixed with 4% PFA in PBS for 10–15 min, followed by three 5-min washes with PBS. For SG Spz, fixed parasites were air-dried in a biosafety hood overnight before proceeding to immunostaining.

Dry-fixed sporozoites and fixed SG-infected HepG2-CD81 cells were blocked and permeabilized with 0.2% Triton X-100/2% BSA in PBS for 20 min at room temperature (RT). Primary antibodies, diluted in 0.2% Triton X-100/2% BSA/PBS, were applied for 1–2 hours at RT or overnight at 4 °C. Samples were washed three times with PBS, then incubated with secondary antibodies for 45 min at RT. After one PBS wash, cells were stained with DAPI for 15 min at RT, followed by three PBS washes and mounting with antifade reagent. Samples were air-dried overnight before imaging.

The primary antibodies used including, *Py*mTIP (1:500, Stefan Kappe, rabbit polyclonal)^56^, *Pb*UIS4 (1:500, goat polyclonal AB0042–500, SIGEN), mNeonGreen (1:400, mouse monoclonal 32F6, Proteintech), Histon H3K9Ac (1:200, mouse monoclonal MABI0305, Gene Tex), *Pv*HSP70 (1:400, rabbit, polyclonal)^7^, *Py*PABP1 (1:200, Scott Lindner, rabbit polyclonal)^38^. Secondary antibodies were used including, donkey anti-mouse 488 (Invitrogen, Cat # A21202; RRID: AB_141607), donkey anti- mouse 594 (Invitrogen, Cat # A21203; RRID: AB_141633), donkey anti-rabbit 488 (Invitrogen, Cat # A-21206; RRID: AB_2532792), donkey anti-rabbit 594 (Invitrogen, Cat # A21207; RRID: AB_141637), donkey anti-rabbit 647 (Invitrogen, Cat # A-31573; RRID: AB_2536183), donkey anti-goat 594 (Invitrogen, Cat # A-11058; RRID: AB_2534105), donkey anti-goat 647 (Invitrogen, Cat # A-21447; RRID: AB_2535864). All the secondary antibodies were used at a dilution 1:1000.

### Microscopy

All fluorescent images were captured using the Stellaris 8 confocal microscopy (Leica Microsystems), equipped with 63x water objective. The Lightning software (Leica Microsystems) was use for image processing. The liver stage counts and sizes were quantified using the ‘Hybrid Duo Count and Measure’ function in Keyence Microscopy (BZ-X710).

### RNA bind-n-seq experiments

For IESI-1, gene sequences that correspond to homologous regions containing the annotated RRM for orthologues from *P. falciparum* (aa 875–1232), *P. vivax* (aa 896–1203) and *P. yoelii* (aa 795–956) were cloned into the pGEX6P1 (Cytiva, cat. no. 28–9546-48) vector for expression with an N-terminal GST-tag. Sequences corresponding to aa 1–109 containing the three CCCH zinc finger domains of IESI-2 orthologues from *P. falciparum, P. vivax* and *P. yoelii* were also cloned downstream of a GST-tag in the pGEX6P1 (Cytiva, cat. no. 28–9546-48) vector for recombinant protein expression in bacteria, *E. coli* Rosetta 2 (DE3)pLysS (EMD Millipore, cat. no. 71401). To prepare for protein expression, bacteria were grown from a starter culture in 100 mL LB broth with 100 μg/mL ampicillin and 35 μg/mL chloramphenicol at 37°C until OD_600_ of approximately 0.6 before induction with 0.2 mM IPTG. Following induction, the temperature was reduced to 30°C and 0.1 mM zinc chloride was added to bacterial cultures expressing IESI-2 and protein was expressed for 2 h before freezing the bacterial pellet at −80°C until purification. Prior to purification, bacteria were lysed in BPER reagent (Thermo Fisher Scientific, cat. no. 89821) at room temperature for 10 min. Purification of the GST-tagged proteins was performed using a GST Fusion Protein purification kit (Genscript, cat. no. L00207) per the manufacturer’s instruction with modifications. The GST-tagged protein purification included the addition of 10 U of DNAse (Biobasic, cat. no. BS88253) and 10 U RNAse A (Biobasic, cat. no. RB0473) to the bacterial lysate. For IESI-2, the pH of the gravity flow buffer was decreased to 6.8 to prevent chelation of the zinc from the CCCH zinc finger in the proteins^57^ before elution at pH 8.0 in the standard elution buffer recommended by the manufacturer. RNA bind-n-seq (RBNS) was carried out at room temperature as in^42^, using 50 nM, 100 nM and 500 nM concentrations of the IESI-1 RRM or IESI-2 zinc finger domains from each species and a 40-mer random *in vitro* transcribed RNA library.

The GST-protein-RNA complexes were pulled down using glutathione high-capacity magnetic agarose beads (Sigma Aldrich, cat. no. G0924) prior to three washes in wash buffer (25 mM Tris, pH 7.5, 150 mM KCl, 60 μg/ml BSA, 0.5 mM EDTA, 0.01% Tween). The RNA was released from the protein complex in elution buffer (10 mM Tris, pH 7.0, 1 mM EDTA, 1%SDS) for 10 min at 70°C and remaining protein was digested with 1.5 μg proteinase K for 15 min at 37°C. The RNA was then purified on Zymo RNA clean and concentrator columns (cat. no. R1016) and eluted into nuclease-free water. The purified RNA and 0.5 pmol of the input library were reverse transcribed using SSIV (Invitrogen, cat. no. 18090010) and sequencing library preparation via PCR with primers and indexes for Illumina Truseq RNA libraries as described in Lambert et al.^58^. RBNS libraries were pooled and single-end sequencing performed with a Miseq V3 at a concentration of 10 pM. Data from RBNS were analyzed in Linux using scripts from the C Burge lab RBNS pipeline to identify sequences enriched the bound RNA (https://github.com/cburgelab/RBNS_pipeline). Representative sequence logos of motifs enriched in RNA-bind-n-seq were generated in seqlogo (DOI: 10.18129/B9.bioc.seqLogo) in R v 4.2.3 and graphs were created in GraphPad Prism v 10.

### Sequence analyses

#### Motif searches

Enriched sequence motifs were identified hypnozoite associated transcripts by using data from (Gural *et al*. 2018)^17^. Transcripts that were increased > 1 Log_2_ Fold Change, *P* < 0.05 in hypnozoites or liver stage parasites were searched for enriched sequenced motifs compared to all *P. vivax* transcripts as background using STREME (https://meme-suite.org/meme/doc/streme.html). To identify occurrences of the RBNS motif in transcripts, a .fasta file containing all *P. vivax* transcribed sequences was searched using SeqKit^59^ v2.10.0 for occurrences of either 5’-TGATGA-3’ or 5’-TGACAC-3’ and the location mapped back to the annotated gene components (intron, exon, UTRs) in the .gtf file for *P. vivax*.

#### Protein Structures

The predicted protein structures of the RRM domain (1,043 aa – 1,123 aa) in PVP01_0939900 and C3H1-type domains (1–100 aa) in PVP01_0604500 were generated using ColabFold^60^ v1.5.5 with default parameters. From ColabFold v1.5.5, the best ranked model was selected and visualized with ChimeraX^61^ v1.9. Sequence conservation was assessed by aligning the corresponding residues in *P. yoelii* of the RRM domain in PY17X_0911200 and CH31-type domains in Py17XNL_000504404.

### Phylogeny

Sequences were retrieved from BLASTP of PVP01_0939900 (IESI-1) & PVP01_0604500 (IESI-2). Sequences were aligned with MAFFT^62^ v7.525 (default parameters) and trimmed with ClipKIT^63^ v2.1.3 using the “smart-gap” mode. The trimmed sequences were then used to construct a maximum likelihood tree (ML) with IQtree^64,65^ v3.0.1. with 1,000 ultrafast bootstrap replicates and the best fit model for IESI-1 (Q.BIRD+F+I+G4) and IESI-2 (Q.MAMMAL+F+I+G4). The *Plasmodium* species *P. gallinaceum* and *P. relictum* were used as outgroups to root both trees and each tree were visualized and exported from ITOL^66^.

## STUDY APPROVAL

This study was carried out in accordance with the recommendations of the NIH Office of Laboratory Animal Welfare standards (OLAW welfare assurance #D16–00119). Mice were maintained under specific pathogen-free conditions at the Center for Global Infectious Disease Research, Seattle Children’s Research Institute. The protocols were approved by the Center for Infectious Disease Research Institutional Animal Care and Use Committee (IACUC) under Protocol SK00505 and SK00666 (for rodent malaria parasites).

## Supplementary Files

This is a list of supplementary files associated with this preprint. Click to download.

- SupplementaryTable1.xlsx
- 6.Sup.FigureFileReduced.pdf
- SupplementaryFileX.xlsx
- SupplementaryFileY.xlsx

**Sup. Figure 1. Structure and conservation of IESI 1 and 2 proteins across diverse parasite species and in hypnozoite transcriptomes. (A)** Comparison of published *P. vivax* hypnozoite transcriptomes. Differentially expressed transcripts from bulk RNA sequencing (Gural et al.^17^, Mancio-silva et al.^18^ and scRNA-seq (Ruberto et al.)^19^ datasets are shown in heatmap with specific examples highlighted. **(B)** Bargraph shows expression of markers of liver stage schizogony (AMA1, GAMA) and IESI-1 and IESI-2 with their relative expression in hypnozoites compared to liver schizonts in *P.cynomolgi* liver-stage transcriptome datasets^20–22^. For each protein, a schematic of the *P. vivax* protein indicates the relative position of the RNA binding domains and any other identifiable domains in the protein (NLS = Nuclear Localization Signal). Red bars indicate regions of homology where color intensity indicates relative sequence identity to the corresponding *P. vivax* protein. Phylogenetic trees show bootstrap values to indicate relative distance between species.

Sup. Figure 2. Generation and validation of ^*uis4*^*Pv*IESI-1^mNeon^ and ^*uis4*^*Pv*IESI-2^mNeon^ transgenic parasite lines.

**(A)** The scheme outlines the creation of ^*uis4*^*Pv*IESI-1^mNeon^ and ^*uis4*^*Pv*IESI-2^mNeon^ lines For each construct, a plasmid containing Cas9, *Py*P230p-specific guide RNA, 5′ and 3′ *Py*P230p homology regions, 5′ and 3′ *Py*UIS4 UTRs, the *Pv*IESI-1 or *Pv*IESI-2 ORF, and mNeon was transfected into the *Py*XNL strain. Transgenic parasites were selected based on DHFR expression. Screening primers are indicated as F and R (Table S1).

**(B–C)** Agarose gel electrophoresis confirming successful integration and overexpression of ^*uis4*^*Pv*IESI-1^mNeon^ **(B)** and ^*uis4*^*Pv*IESI-2^mNeon^ **(C)** using recombinant-specific primers (labelled as F and R) as described in Table S1.

Sup. Figure 3. Generation and validation of ^uis4^*Pv*IESI-1, ^*uis4*^*P*vIESI-2, and ^*uis4*^mNeon transgenic parasite lines.

**(A)** Schematic representation of the constructs used to generate ^*uis4*^*Pv*IESI-1, ^*uis4*^*Pv*IESI-2 parasite lines were generated. A Cas9-expressing plasmid containing the *PyP230p* guide RNA, 5′/3′ *PyP230p* homology regions, 5′/3′ *PyUIS4* UTRs, and either the *Pv*IESI-1 or *Pv*IESI-2 ORF (without mNeon) was transfected into *PyXNL* parasites.

**(B)** For uis4mNeon, a similar construct was used, replacing the *Pv*IESI-1 or *Pv*IESI-2 ORF with mNeon.

**(C–E)** PCR verification of transgenic lines using primers F and R (Table S1) confirming successful integration and overexpression of ^uis4^*Pv*IESI-1, ^uis4^*Pv*IESI-2, and *uis4*mNeon. Transgenic parasites were selected based on DHFR expression.

**Sup. Figure 4. (A)** No significant differences in liver parasite size were observed at 6 hpi. **(B**) The graph illustrates liver sizes across strains (*Py*XNL, ^*uis4*^mNeon, ^*uis4*^*Pv*IESI-1, ^*uis4*^*Pv*IESI-2). Error bars represent the standard deviation from three independent experiments. The mNeon signal gradually decreased as parasite size increased in ^*uis4*^*Pv*IESI-1^mNeon^ line **(C)** and ^*uis4*^*Pv*IESI-2^mNeon^ line **(D)**. Conversely, the mNeon signal remained stable in ^*uis4*^mNeon parasites **(E)**. Scale bar, 10 μm.

**Sup. Figure 5. Determining the *in vitro* RNA binding specificity of IESI-1 and IESI-2 across species. (A, D)** Results from RNA-bind-n-seq show the top motifs enriched above occurrences of all possible 6-mers as R-values for *P. falciparum* and *P. yoelii* IESI-1 and IESI-2 respectively. **(B, E)** Representative sequence logo of 10 top motifs enriched in RNA-bind-n-seq was generated in seqlogo in R for for *P. falciparum* and *P. yoelii* IESI-1 **(B)** and IESI-2 **(E)**. Correlation between the average enrichment (R-values) obtained for motifs in the *P. falciparum* and *P. vivax* IESI-1 **(C)** and IESI-2 **(F)** experiments with top motifs highlighted in color.
